# Gone with the currents: lack of genetic differentiation at the circum-continental scale in the Antarctic krill *Euphausia superba*

**DOI:** 10.1186/1471-2156-12-32

**Published:** 2011-04-12

**Authors:** Erica Bortolotto, Ann Bucklin, Massimo Mezzavilla, Lorenzo Zane, Tomaso Patarnello

**Affiliations:** 1 Department of Biology, University of Padova, via U. Bassi 58B -35121 Padova, Italy; 2 Department of Marine Sciences, University of Connecticut, Groton CT 06340 USA; 3 Department of Public Health, Comparative Pathology and Veterinary Hygiene, University of Padova, Viale dell'Università 16, 35020 Legnaro (PD), Italy

## Abstract

**Background:**

Southern Ocean fauna represent a significant amount of global biodiversity, whose origin may be linked to glacial cycles determining local extinction/eradication with ice advance, survival of refugee populations and post-glacial re-colonization. This pattern implies high potential for differentiation in benthic shelf species with limited dispersal, yet consequences for pelagic organisms are less clear. The present study investigates levels of genetic variation and population structure of the Antarctic krill *Euphausia superba *using mitochondrial DNA and EST-linked microsatellite markers for an unprecedentedly comprehensive sampling of its populations over a circum-Antarctic range.

**Results:**

MtDNA (ND1) sequences and EST-linked microsatellite markers indicated no clear sign of genetic structure among populations over large geographic scales, despite considerable power to detect differences inferred from forward-time simulations. Based on ND1, few instances of genetic heterogeneity, not significant after correction for multiple tests, were detected between geographic or temporal samples. Neutrality tests and mismatch distribution based on mtDNA sequences revealed strong evidence of past population expansion. Significant positive values of the parameter *g *(a measure of population growth) were obtained from microsatellite markers using a coalescent-based genealogical method and suggested a recent start (60 000 - 40 000 years ago) for the expansion.

**Conclusions:**

The results provide evidence of lack of genetic heterogeneity of Antarctic krill at large geographic scales and unequivocal support for recent population expansion. Lack of genetic structuring likely reflects the tight link between krill and circum-Antarctic ocean currents and is consistent with the hypothesis that differentiation processes in Antarctic species are largely influenced by dispersal potential, whereas small-scale spatial and temporal differentiation might be due to local conditions leading to genetic patchiness. The signal of recent population growth suggests differential impact of glacial cycles on pelagic Antarctic species, which experienced population expansion during glaciations with increased available habitat, *versus *sedentary benthic shelf species.

EST-linked microsatellites provide new perspectives to complement the results based on mtDNA and suggest that data-mining of EST libraries will be a useful approach to facilitate use of microsatellites for additional species.

## Background

Antarctic waters host a unique cold-adapted fauna that constitutes a significant amount of the global biodiversity [1] and has been the subject of biological studies for over a century. Starting with the early Antarctic expeditions of the R.R.S. Discovery (1925-1927) and H.M.S. Challenger (1872-1876), scientists have concentrated their efforts first on the collection and identification of Antarctic organisms, and thereafter on the investigation of how they are spatially distributed, leading to the description of Antarctic biogeography [2,3]. In addition to the considerable work on the biology of Antarctic marine fauna and estimates of Antarctic biodiversity in general [4-7], recent studies have focused on understanding the genetic patterns, speciation processes, and/or environmental mechanisms that have lead to recent Antarctic biodiversity patterns [8-15]. In particular, most studies in the past two decades focused on Antarctic marine benthos, for which many new taxa and cryptic species have been described in the costal shelf [16,17], whereas recent work in the deep sea has documented, by means of molecular tools, a spectacular and previously-unsuspected diversity [18].

Based mainly on studies of benthos, hypotheses have been formulated to explain Southern Ocean faunal distribution and Antarctic endemism in relation to the influences of oceanographic processes (e.g., Antarctic Convergence and Antarctic Polar Front, ice advance and retreats during glaciations as evidenced by paleoclimatic studies) in shaping the evolutionary history of Antarctic species [19,20]. The recent finding that many of the benthic species probably survived the last glacial maximum [16,21] implies processes of local extinction/eradication and survival of refugee populations in suitable areas, followed by post-glacial re-colonization. This pattern implies high potential for differentiation, particularly in species with limited dispersal, such as benthic brooders, but several exceptions seem to show the possibility of genetic differentiation, including invertebrates with pelagic larvae, such as the crinoid *Promachocrinus kerguelensis *[22]. Significant differentiation among populations has been found in several species of nototheniod fish, all characterized by pelagic larvae. Signals for restricted gene flow were reported not only in demersal notothenioids but also in the few existing pelagic species of this fish sub-order (reviewed in [23]). This suggests that the interplay between life history traits (i.e., larval duration, philopatry) and marine currents may contribute to isolating populations and ultimately lead to allopatric speciation. From this perspective, further investigation of species with a high dispersal potential (e.g., pelagic/planktonic forms) is critically needed to assess whether, on the circum-continental scale, this trait results in a genetic homogenization of the gene pool preventing further evolution.

Antarctic krill (*Euphausia superba*) is a holoplanktonic species that is probably the most abundant multicellular organism on the planet [24], with a biomass estimation of 44 million tonnes in the Southwest Atlantic sector of the Southern Ocean [25]. *Euphausia superba *is one of the best-studied pelagic invertebrates, with numerous studies since the 1920s and 1930s [26,27] and a key species in the Southern Ocean's marine ecosystem. It occupies a central role in the Antarctic food web as the main food source for Antarctic fish, penguins, pelagic seabirds, seals, and whales, and also sustains the largest single-species crustacean fishery in the world [28].

*Euphausia superba *has a circumpolar distribution and shows typical swarming behaviour that leads to local aggregations and produces a typical patchy distribution. Although very few large-scale studies have shown obvious correlation between krill distribution and current systems (but see [27]), it has been suggested that large-scale, heterogeneous distributions could be caused by the gyral circulation pattern of Southern Ocean water masses [29,30]. In fact, areas of higher adult krill concentration are associated with the major oceanic gyres, which are formed by interactions between the two main surface currents: the eastward-flowing Antarctic Circumpolar Current (ACC) and the westward-flowing Coastal Current (CC). These currents play an important role in krill dispersal, since both adults and larvae can be passively transported among areas of high krill abundance.

To ensure the sustainable harvesting of krill in the Southern Ocean, CCAMLR (Convention on the Conservation of Antarctic Marine Living Resources) was designed in 1981 to set precautionary catch limits. For the Southwest Atlantic sector, the limit was 1.5 million tons per year. Sound management of the krill fishery requires fundamental knowledge of krill distribution; accurate estimation of abundance, biomass, and flux among areas; detailed understanding of life history; and characterization of genetic differentiation among populations.

Unfortunately, Southern Ocean krill have proven to be an unyielding organism for the isolation of high-resolution genetic markers, such as microsatellites [31,32]. Early studies using allozymic allelic frequencies indicated genetic homogeneity across the entire distribution range, with several exceptions [33-35]. More recently, Zane et al. [36] studied the genetic structure of *E. superba *using a portion of mitochondrial NADH dehydrogenase gene (ND1) and found weak but significant genetic differentiation (Φst = 0.0213; p < 0.05) among samples, based on rather limited sampling in the Weddell Sea and near South Georgia. The authors hypothesized that Weddell Sea gyre and/or the Weddell-Scotia Confluence could be responsible for the reduced gene flow between these two areas. Nonetheless, the possibility that the observed genetic differentiation could result from temporal (rather than spatial) variability could not be excluded, since the samples were collected in different years. Evidence of small-spatial scale genetic heterogeneity, based on SNPs (Single Nucleotide Polymorphisms) in mitochondrial cytochrome *b*, has been reported by Batta-Lona et al. [37] among samples and life stages of *E. superba *in the Western Antarctic Peninsula (WAP). In contrast, no genetic differences in haplotype frequencies for mitochondrial cytochrome c oxidase subunit I (COI) were reported among swarms of *E. superba *collected at the local scale [38], although COI did reveal heterogeneity among samples of *E. crystallorophias *(a sister species to *E. superba*) collected from the same region [39]. This latter suggests that differences among krill swarms or perhaps cohorts might explain part of the observed genetic differentiation, especially at small- to regional geographic scales. It is therefore important that population genetics studies in general - and for krill in particular - investigate genetic variation associated with a range of both geographic and temporal scales, as also proposed by Siegel [26]. In addition, genetic differentiation among krill stocks in different regions should consider biological processes (e.g., life stages, population cohorts, and swarms), as proposed by Batta-Lona et al. [37] and should be based upon multiple samples collected from each region.

The present study extends the analysis of the population structure of *E. superba *by taking into account samples collected from different areas and in different years. Mitochondrial DNA sequencing (ND1) and three microsatellite markers obtained by data mining from an *E. superba *cDNA library [40] were employed as molecular markers. New mitochondrial sequence information was obtained and combined with the mitochondrial data from Zane et al. [36] in order to study both spatial and temporal variability of krill genetic structure. Mitochondrial data were also analyzed to determine whether the Southern Ocean krill species population has experienced fluctuations in size at evolutionary time scales, and also to amend previous estimates of population expansion. Microsatellites data were included to increase the analytical power, since these markers are known to be more polymorphic than mtDNA [41]. This study describes new scenarios for krill population dynamics and structure based upon analysis of the largest and most extensive collection of *E. superba *DNA analyzed to date.

## Methods

### Sample collection and DNA extraction

**Figure 1:**
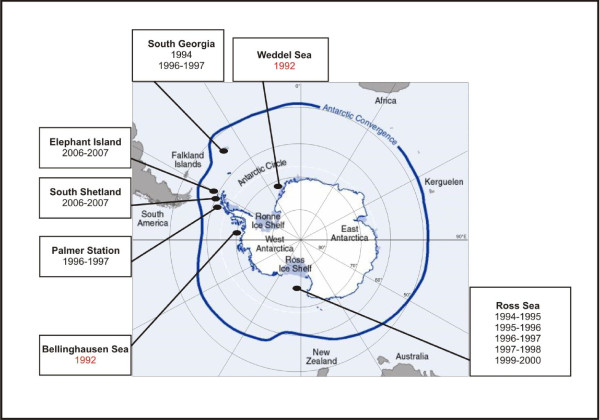
**Map of the sampling sites of population samples analysed in this study**. Samples from Bellinghausen Sea and Weddell Sea were used only for the analysis of mitochondrial variability.

Samples analysed in this study were obtained during several cruises to the Southwest Atlantic and Pacific (Ross Sea) sectors of the Southern Ocean (Table 1; Figure 1). For the analysis of mitochondrial variability, sequences were obtained for 8 new population samples and merged with previously published data [36], yielding a total of 12 population samples. Genetic analyses using nuclear loci were performed using 10 population samples (DNA from B-1992 and W-1992 samples were no longer available; Table 1).

**Table 1:** Site of collection, year of collection, sample size, source and abbreviation for each population.

Population	Austral summer	Sample Size	Source	Code
South Georgia I.^(*)^	1993-1994	70	BAS (GFC JR06)	SG-1994

South Georgia I.	1996-1997	60	BAS (GFC JR26)	SG-1997

Elephant Island	2006-2007	48	AWI (PS ANT XXIII/8)	EI-2007

South Shetland I.	2006-2007	48	AWI (PS ANT XXIII/8)	SS-2007

Weddel Sea^(*+)^	1991-1992	68	AWI (PS ANT X/8)	W-1992

Bellingshausen Sea^(*+)^	1991-1992	63	BAS (GFC JR02)	B-1992

Palmer Station	1996-1997	54	BAS (GFC JR26)	P-1997

Ross Sea^(*)^	1994-1995	48	PNRA (X expedition)	R-1994

Ross Sea	1995-1996	60	PNRA (XI expedition)	R-1995

Ross Sea	1996-1997	45	PNRA (XII expedition)	R-1996

Ross Sea	1997-1998	48	PNRA (XIII expedition)	R-1997

Ross Sea	1999-2000	48	PNRA XV (expedition)	R-1999

^(*) ^Population samples analysed in [36] for mtDNA variation.

^(+) ^Population samples no more available for nuclear DNA analysis.

Total genomic DNA was extracted from 5 - 10 mg of muscle tissue of each individual using a salting-out protocol [42]. The DNA solution was stored at -20°C before PCR amplification.

### Molecular population genetic analysis of mitochondrial DNA

A ~200 base-pair portion of NADH dehydrogenase sub-unit 1 (ND1) was amplified using specific primers for *E. superba: ND1aR, 5'*-*GGCAAAAATCTTTTCCAGGCTAAGTA*-3', and *ND1F, 5'*-*TTTTTCTTGTGTTGTACTAGTTTAGG-*3' [36]. One μl of total genomic DNA was used in 10 μl of reaction mix (50 mM KCl, 10 mM Tris-HCl, 0.1% TritonX-100, 1.5 mM MgCl_2_, 17.5 μM of each dNTPs and 0.5 μM of each primer) with 0.2 unit of Taq DNA polymerase (Promega, Madison, Wisconsin, USA). The thermal cycler program was: (i) pre-denaturation at 94°C for 2 min; (ii) 35 cycles of denaturation at 94°C for 30 sec, annealing at 49°C for 30 sec, extension at 72°C for 40 sec; and (iii) final extension for 5 min at 72°C.

The fragments were sequenced using the ND1aR primer in an automated DNA sequencer (ABI 3100 or 3700; Applied Biosystems Inc., Foster City, California, USA) after enzymatic purification. Chromatograms were examined, manually edited using ChomasPro 1.41 (Technelysium Pty) and aligned using GENEDOC 2.6.002 [43]. Aligned sequences were combined with published data from Zane et al. [36] and analyzed together.

Genealogical relationships among sequences were reconstructed using the TCS program [44], which calculates the frequency of each haplotype in the sample and uses parsimony to create a network of haplotypes; the default 95% cut-off was used.

Analyses of genetic variability, differentiation among populations, deviation from mutation-drift equilibrium, and historical demography were performed using Arlequin 3.11 [45] and DNAsp 4.50.3 [46]. Genetic variability was measured by haplotype diversity (*h*) and nucleotide diversity (*π*; [47]).

Population genetic structure was investigated by Analysis of MOlecular VAriance, (AMOVA; [48]), which partitions the total variance into covariance components and verifies the hierarchical or non-hierarchical distribution of the variability as follows: among populations (fixation index *Φst*), among populations within groups (*Φsc*), and among groups (*Φct*). The analyses were performed using Kimura-2-Parameter molecular distances [49]; given the limited divergence between haplotypes, very similar results were obtained using different distances. The statistical significance of the fixation indices was assessed under the null hypothesis of panmixia by performing 10,000 permutations of the original data set by random reallocation of individuals to each population and of populations to each group. In addition, pairwise *Φst *values were calculated between all populations and the significance tested as above.

Neutrality tests and mismatch distribution analysis were used to investigate signatures of past demographic events that affected the populations. Neutrality tests, which were developed to test the neutral hypothesis under an infinite-site model with constant population size [50], were used to infer past population size based upon deviation from the mutation-drift equilibrium [51]. In particular, Tajima's *D *[[51], Fu's *Fs *[52] and Rozas's *R_2 _*[53] tests were performed. Tajima's *D *test calculates the value of *D *(i.e., the ratio of two different estimations of the parameter *θ*), which for mtDNA is twice the product of the effective population size and the substitution rate; *D *is expected to be negative if the population has experienced an expansion. Fu's *Fs *test evaluates the probability of observing a random neutral sample with the same or fewer alleles than the observed value, assuming the same number of pairwise differences (an estimator of *θ*). The *F_S _*statistic is very sensitive to population demographic expansion, which generally leads to large negative *F_S _*values. Rozas's *R_2 _*test considers the difference between the number of the singleton substitutions and the average number of nucleotide differences. This statistic is appropriate to distinguish population growth from constant size population; lower values of *R_2 _*are expected under a population expansion event and *R_2 _*is more powerful than Fu's *F_S _*for small sample sizes.

Rogers and Harpending [54] showed that demographic events in populations affect the distribution of the observed pairwise differences between haplotypes or DNA sequences, which they termed the mismatch distribution. For a population at equilibrium of size, with no recombination and under an infinite site model, the mismatch curve fits a Poisson distribution. For a population that has undergone demographic expansion, the distribution of pairwise differences between haplotypes assumes a unimodal curve. The model assumes an initial population size *θ_0 _*at time 0 (*θ_0 _= 2N_0_u*) that grows to a new size *θ_1 _*(*θ_1 _= 2N_1_u*), where *N *is the population size and *u *is the substitution rate for the whole haplotype. The position of the mean (*τ*) in the mismatch distribution is used to infer the start of the expansion by the equation *τ = 2ut*, where *u *is the substitution rate for the whole haplotype and *t *is the time.

### Molecular population genetic analysis of microsatellite markers

An Expressed Sequence Tag (EST) database specific for *E. superba *[40] was mined to identify microsatellite or Simple Sequence Repeats (SSRs) markers in transcripts. A total of 1,770 ESTs was examined using MISA (MIcroSAtellite identification tool) software [55] and Tandem Repeat Finder [56] to detect tandemly repeated sequences. To maximize the number of informative markers, the search included compound and interrupted microsatellites of any SSR motif, excluding mononucleotide stretches. SSRs containing sequences with enough flanking region were selected and primers were designed using PRIMER3 [57]. One μl of total genomic DNA was used for each amplification, using the same PCR mix described for mitochondrial markers. The thermal cycler profile was: (i) pre-denaturation at 94°C for 2 min; (ii) 35 cycles of denaturation at 94°C for 30 sec, annealing at 55 or 52°C for 30 sec, extension at 72°C for 30 s; and (iii) final extension for 5 min at 72°C. Forward primers were labelled with different fluorescent dyes and a fraction of the PCR product was loaded on an ABI PRISM 3100 or 3700 automated DNA sequencer. ROX-400 was used as size standard. Allele sizes were assigned using GENOTYPER 3.7 (Applied Biosystems Inc., Foster City, California, USA).

Departures from Hardy-Weinberg and linkage equilibrium were tested with the exact test implemented in the software GENEPOP[58]. Genetic population structure and temporal stability were investigated as before by hierarchical and non-hierarchical AMOVA and pairwise *Fst *statistics, implemented in Arlequin 3.01 [48]. FreeNA [59] was used to test the occurrence of null alleles and estimate their frequencies for each locus and population analyzed based upon the Expectation Maximization (EM) algorithm [60].

Estimation of long-term effective population size was conducted using the Bayesian method in LAMARC 2.1.3. [61], a Markov chain Monte Carlo coalescent genealogy sampler. The program estimates *θ*= 4*N_e_μ *(where *N_e _*is the effective population size and *μ *is the nuclear mutation rate per generation), together with the exponential growth rate *g*. The parameter *g *is the exponent of the exponential growth rate formula that gives the *θ *at a time *t *in terms of the modern-day *θ *and the growth rate: *θ (t) = θ e^-gt^*. Ten initial brief chains (to get the driving values) and two final chains (to narrow the final values) with 20 million genealogies were generated; sampling was done every 1,000 genealogies, with a burn-in of 1,000 iterations. Mutation was modeled using a Brownian-motion approximation to the stepwise model [62]. Bayesian priors for *θ *and *g *were drawn from uniform distributions (1^-5^<*θ *< 100; -5,000 < g < 10,000). In order to increase the maximum allowed *θ *for the data set, the relative mutation rate of each locus was set to 150. TRACER 1.4 was used to check the convergence of the chains [63]. Estimates of *θ *and *g *were re-scaled and the effective population size (*N_e_*) was estimated from *θ *using the equation *N_e_= θ/4 μ *and assuming a mutation rate of *μ *= 5 × 10^-4 ^per generation, which was intermediate between two widely-used estimates: 10^-3 ^and 10^-4 ^(the former extremely high, the latter too conservative [64,65]).

### Forward-time genetic simulations and assessment of statistical power

The statistical power of our dataset was tested in order to establish the extent to which nuclear and mitochondrial data are able to detect genetic heterogeneity between samples. To this end, we simulated populations at a known level of divergence, performed non-hierarchical AMOVA on replicates of the simulated dataset (N = 100) and recorded the fraction of simulated populations for which genetic homogeneity was rejected (α = 0.05). Simulations were carried out using simuPOP [66], a flexible forward-time population genetics simulation environment that allows generation of individuals characterized by both diploid genotypes (microsatellite loci) and sequence haplotypes (mitochondrial DNA fragment) and provides appropriate mutation models and inheritance transmission schemes for both kind of markers.

In particular, 10 populations were simulated, corresponding to samples for which both nuclear and mitochondrial data were available (Table 1). Sex ratio was assumed to be 1:1 and estimates of long-term effective population size (see above) were used to set the population size parameter to N = 40,000. A multilocus genotype for 3 diploid loci and a sequence haplotype for 1 haploid locus was assigned to each simulated individual, based on the frequencies of microsatellite alleles and ND1 sequences observed in the empirical dataset. A stepwise mutation model with a rate of 5 × 10^-4 ^mutations per generation was assumed for microsatellites [65], while for mtDNA a K-allele model was used with a locus substitution rate of 3 × 10^-6^, resulting from the canonical 1% substitutions per site per million years [38] and locus sequence length of approximately 154 bp, with a generation time of approximately 2 years [36]. Individuals were allowed to sexually reproduce with a random mating scheme for a predefined number of generations; at each generation parents died after reproduction and offspring received their alleles by mendelian inheritance and the mother mtDNA haplotype by clonal transmission. At the end of each simulation, the appropriate number of multilocus genotypes and haplotypes (matching samples size of the empirical dataset) was collected from each simulated population, formatted separately for the diploid and haploid loci, and analysed with Arlequin v 3.01, with the same settings used for the empirical dataset. *Fst*/*Φst *values and their significances were recorded.

Simulations were performed under a pure drift model, with the effective number of migrants per generation (*Nm*) set to 0 using an approach similar to Ryman and Palm [67], and by setting *Nm *= 1 and 5, corresponding to thresholds proposed to define separate populations [68]. The populations were allowed to diverge for 100, 200, 300, and 400 generations to generate *Fst *values ranging from 0.001 to 0.004 for microsatellites and from 0.004 to 0.02 for mtDNA. For a each of the 12 set of parameters simulated (corresponding to 3 migration regimes × 4 drifting amounts), 100 replicates were performed and the fraction of significant *Fst*/*Φst *values obtained from replicated simulations was taken as an estimate of the power to detect heterogeneity under the specific scenario.

## Results

### Mitochondrial DNA

The new sequence analysis for 393 specimens obtained in this study was combined with published data from Zane et al. [36], yielding a new data set of 641 sequences of 154 base pairs from 12 population samples. All sequences have been submitted to GenBank (Additional file 1, Table S1). Thirty-six new haplotypes were detected in the new population samples, for a total of 124 haplotypes in the combined dataset. Analysis of polymorphic sites implemented by DnaSP [46] identified 46 variable sites (18 of which were singletons that occurred in a single individual) and 28 parsimony informative sites. Some of these sites showed multiple substitutions; for the entire dataset, a total of 59 substitutions was detected. Considering synonymous and replacement changes using *Drosophila *mtDNA code, 3 singleton substitutions were detected that caused an amino acid change: from Alanine to Serine (position 105); from Serine to Cysteine (position 120); and from Valine to Isoleucine (position 134). The average value of haplotype diversity (*h*) was 0.8561 (range from 0.8271 to 0.8889), while average value of nucleotide diversity (π) was 0.013937 (range from 0.01196 to 0.01642; Table 2).

**Table 2:** Haplotype diversity (*h*) and nucleotide diversity (π) calculated for each population.

Population	Population size	Haplotype number	**Haplotype diversity *h***^(*)^	**Nucleotide Diversity π**^(*)^
**SG-1994**	70	27	0.8232 (0.0425)	0.01365 (0.0085)

**SG-1997**	60	30	0.8853 (0.0345)	0.01642 (0.0099)

**EI-2007**	47	20	0.8779 (0.0380)	0.01400 (0.0087)

**SS-2007**	47	18	0.8779 (0.0343)	0.01428 (0.0089)

**W-1992**	68	22	0.8889 (0.0242)	0.01372 (0.0085)

**B-1992**	63	22	0.8525 (0.0365)	0.01391 (0.0086)

**P-1997**	50	15	0.8229 (0.0402)	0.01196 (0.0077)

**R-1994**	47	21	0.8696 (0.0419)	0.01430 (0.0089)

**R-1995**	60	21	0.8271 (0.0420)	0.01329 (0.0083)

**R-1996**	40	17	0.8641 (0.0416)	0.01470 (0.0091)

**R-1997**	44	18	0.8626 (0.0423)	0.01352 (0.0085)

**R-1999**	45	17	0.8788 (0.0364)	0.01378 (0.0086)

**Total**	641	124	h = 0.8561 (0.0111)	π = 0.013937 (0.0086)

^(*) ^Standard deviation is reported between brackets.

**Figure 2:**
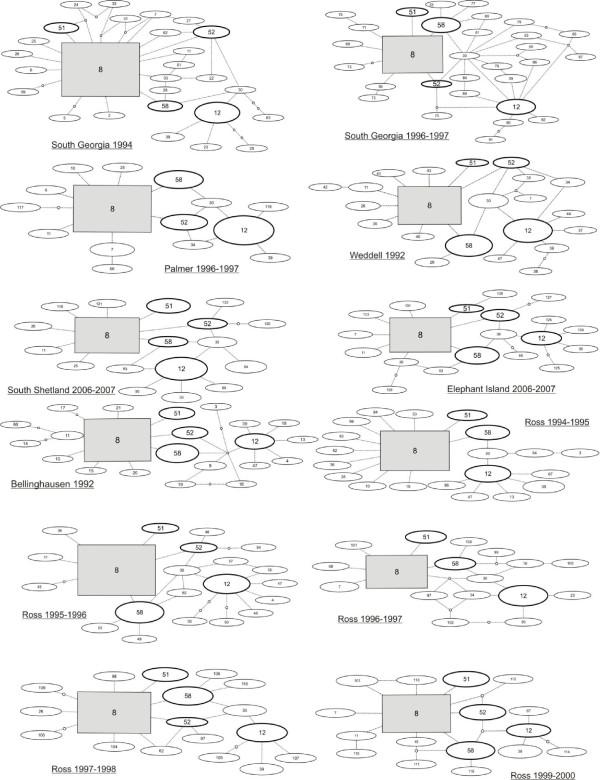
**ND1 haplotype network for each population**. The most frequent haplotypes are reported in grey boxes (8) and in thick ovals (12, 51, 52 and 58). Size of boxes and ovals is proportional to the frequency of each haplotype.

A network representation was used to infer haplotype genealogies using TCS [44]. The high number of rare haplotypes produced a complex cladogram; in order to avoid this complexity, networks are reported for each population sample (Figure 2). The distribution of haplotypes in the populations did not show evidence of geographic structure. In fact, frequent haplotypes (e.g. 8, 12, 51, 52 and 58) were shared among all the populations and differences were due only to rare haplotypes, often present in a single individual (Additional file 1, Table S1).

Analysis of molecular variance (AMOVA) [48] was performed using both non-hierarchical (i.e., all populations in one group) and hierarchical designs (i.e., populations subdivided into two geographical groups corresponding to Southern Ocean South Atlantic and Pacific sectors; Table 3). All the variation (100%) was due to within-population differences and no evidence for subdivision into genetically distinct populations or groups of populations was found. Genetic homogeneity was also confirmed by negative or close to zero pairwise *Φst *values; P-values were not significant after Bonferroni correction (Table 4). As in the previous study by Zane et al. [36], pairwise *Φst *values for comparisons between samples of Weddell Sea and South Georgia 1994 were significant at the P = 0.05 level (*Φst *= 0.02130, P = 0.03320). A similar result was obtained in this study between two temporal replicates from South Georgia (*Φst *= 0.02438, P = 0.02930).

**Table 3:** Analysis of molecular variance (AMOVA) of ND1.

	Source of Variation	Variance Components	Fixation Index	P value
**One group of 12 populations**	**Among Populations**	-0.00255 Va	*Φst*: -0.00230	0.70
	**Within Populations**	1.10738 Vb		

**Two groups** **Atlantic Pacific sector**^(*)^	**Among Groups**	-0.00101 Va	*Φct*: -0.00091	0.68
	**Among Populations Within Groups**	-0.00203 Vb	*Φsc*: -0.00184	0.63
	**Within Populations**	1.10738 Vc	*Φst*: -0.00275	0.70

AMOVA based on Kimura two parameters molecular distance among haplotypes with significance calculated by 10,000 permutations of the original dataset.

^(*) ^Geographical groups: one group with samples from Southern Ocean Atlantic Sector (SG-1994, SG-1997, EI-2007, SS-2007, W-1992, B-1992, P-1997) and the other with samples from the Southern Ocean Pacific Sector (five temporal replicates from Ross Sea: R-1994, R-1995, R-1996, R-1997, R-1999).

**Table 4:** Pairwise *Φst *for ND1 (below the diagonal) and associated P-values (above the diagonal).

	SG-1994	SG-1997	EI-2007	SS-2007	W-1992	B-1992	P-1997	R-1994	R-1995	R-1996	R-1997	R-1999
**SG-1994**	*	0.02930	0.66699	0.11133	0.03320	0.46680	0.35254	0.30078	0.11133	0.18652	0.42383	0.66797

**SG-1997**	0.02438	*	0.38965	0.56738	0.90137	0.15625	0.23438	0.68945	0.52441	0.77832	0.29980	0.11035

**EI-2007**	-0.00592	-0.00109	*	0.45312	0.42188	0.94238	0.63379	0.92480	0.84863	0.84277	0.98438	0.99707

**SS-2007**	0.01407	-0.00510	-0.00315	*	0.79785	0.29102	0.46094	0.86328	0.53516	0.91504	0.57227	0.21289

**W-1992**	0.02130	-0.00852	-0.00203	-0.00893	*	0.19824	0.34766	0.81445	0.75488	0.62891	0.41309	0.12012

**B-1992**	-0.00195	0.00798	-0.01136	0.00172	0.00505	*	0.43066	0.60742	0.59961	0.47070	0.88477	0.78320

**P-1997**	-0.00023	0.00474	-0.00778	-0.00482	0.00011	-0.00214	*	0.58008	0.40234	0.73535	0.44824	0.31738

**R-1994**	0.00186	-0.00752	-0.01271	-0.01229	-0.00950	-0.00609	-0.00696	*	0.89746	0.95117	0.97168	0.52148

**R-1995**	0.01061	-0.00382	-0.01003	-0.00527	-0.00737	-0.00468	-0.00249	-0.01092	*	0.70215	0.88867	0.34082

**R-1996**	0.00707	-0.00940	-0.01171	-0.01477	-0.00717	-0.00347	-0.01044	-0.01579	-0.00840	*	0.79590	0.45020

**R-1997**	-0.00268	0.00083	-0.01477	-0.00643	-0.00165	-0.01052	-0.00405	-0.01558	-0.01133	-0.01217	*	0.87402

**R-1999**	-0.00625	0.01375	-0.01689	0.00757	0.01280	-0.00872	0.00079	-0.00519	0.00128	-0.00274	-0.01245	*

*Φst *calculated using Kimura two parameters molecular distance and P-values based on 10000 permutations of the original dataset; significance threshold α = 0.000758 after correction for multiple comparisons.

Results of neutrality tests and associated P-values are reported in Table 5. Both Tajima's *D *[51] and Fu's *Fs *[52] tests assumed negative values in each population. Fu's *Fs *and *R_2 _*[53] associated probabilities were highly significant in each population. When pooling samples together, Tajima's *D *provided strong support for the departure from mutation-drift equilibrium (Table 5).

**Table 5:** Neutrality tests calculated for each population and for one group including all the populations.

Population	Tajima's D	Fu's Fs	Rozas's R_2_
**SG-1994**	-1.40664 P = 0.07422	-24.07266 p < 0.00001	0.16190 p < 0.00001

**SG-1997**	-1.27269 P = 0.10043	-26.64092 p < 0.00001	0.16062 p < 0.00001

**EI-2007**	-1.26528 P = 0.1029	-14.4701 p < 0.00001	0.16125 p < 0.00001

**SS-2007**	-1.09166 P = 0.14322	-10.86864 p < 0.00001	0.16102 p < 0.00001

**W-1992**	-1.08457 P = 0.14377	-15.06492 p < 0.00001	0.16169 p < 0.00001

**B-1992**	-1.60901 P = 0.04433	-15.49807 p < 0.00001	0.16195 p < 0.00001

**P-1997**	-1.09406 P = 0.14237	-7.8086 p < 0.00001	0.16216 p < 0.00001

**R-1994**	-1.08828 P = 0.14408	-16.0212 p < 0.00001	0.16320 p < 0.00001

**R-1995**	-1.32986 P = 0.083	-14.82281 p < 0.00001	0.16131 p < 0.00001

**R-1996**	-0.8087 P = 0.22	-10.14513 p < 0.00001	0.16117 p < 0.00001

**R-1997**	-1.24047 P = 0.10844	-11.95712 p < 0.00001	0.16042 p < 0.00001

**R-1999**	-1.04076 P = 0.15658	-9.98313 p < 0.00001	0.16177 p < 0.00001

**Total**	-1.79083 P = 0.01961	-26.28904 p < 0.00001	0.16083 p < 0.00001

**Figure 3:**
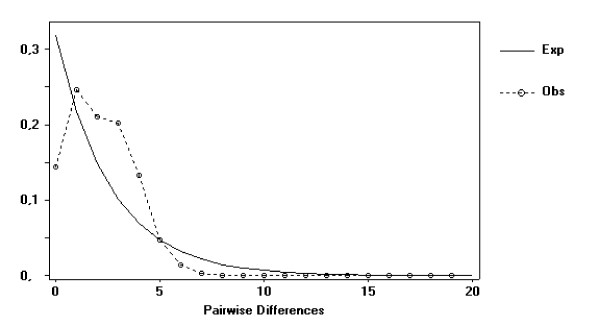
**Distribution of number of differences between pairs of haplotypes observed in *E. superb*a**. Unbroken line represents the hypothetical distribution curve for a population at equilibrium of size, while broken line represents the curve obtained by observed data.

The past demographic history was further investigated by mismatch distribution. The observed data distribution was unimodal (Figure 3), indicating that the population experienced a demographic expansion in the past [29], with a peak at τ = 2.496 and confidence interval (CI) = 0.438-5.607.

### Microsatellite markers

Among 1,170 ESTs [40], 68 sequences contained di-, tri-, tetra-, penta- and esanucleotide repeats that are potentially useful for population genetics analysis. As expected for coding sequences, the most common repeat module found was the trinucleotide, with different combinations of pure, compound and interrupted microsatellites. Many ESTs were discarded because they presented microsatellites with long repeats interrupted by up to 100 bp. Only 12 sequences with enough flanking regions for appropriate primer design were found. For each of these 12 sequences, three or four primers were designed and tested on 8 individuals to increase the probability of obtaining good amplifications with clear and unique amplified products (Additional file 2, Table S2). Due to an unexpectedly high rate of unsuccessful amplification, only seven loci were selected for preliminary genotyping and forward primers were labelled with fluorescent dyes. Preliminary genotyping analysis of the selected microsatellite markers detected two monomorphic loci, which were excluded. Two additional loci were discarded that were characterized by an unusual profile, with more than two alleles for each individual. The remaining three loci were used for population genetic analysis (Table 6). These were shown to be in linkage equilibrium. Locus A_11 _and B_22 _were in Hardy-Weinberg equilibrium for each population sample; low probabilities associated with the exact test (P_HW _< 0.001) indicated strong disequilibrium at locus N_11 _for each sample. Since disequilibrium was detected only for this locus, the occurrence of null alleles appeared to be the most probable explanation and allelic and genotypic frequencies were re-calculated for locus N_11 _using FreeNA [59].

**Table 6:** Population characteristics based on three microsatellite markers.

Population	Population Size	Locus	N° alleles	Allelic Richness	H_o_^(*)^	H_e_^(+)^	P_HW_^(x)^
**SG-1994**	49	A_11_	7	6.020	0.714286	0.718073	0.4094

		B_22_	4	3.934	0.244898	0.2266	1.0000

		N_11_	20	19.112	0.230769	0.926741	<0.001

**SG-1997**	48	A_11_	7	6.318	0.659574	0.71883	0.9010

		B_22_	5	4.062	0.291667	0.3353	0.5323

		N_11_	24	21.863	0.318182	0.93887	<0.001

**EI-2007**	48	A_11_	7	6.497	0.708333	0.72456	0.6937

		B_22_	4	3.972	0.416667	0.4156	0.2414

		N_11_	18	16.983	0.358974	0.911087	<0.001

**SS-2007**	48	A_11_	7	6.402	0.787234	0.747426	0.9318

		B_22_	4	3.592	0.270833	0.2594	0.4117

		N_11_	22	20.127	0.365854	0.929237	<0.001

**P-1997**	54	A_11_	6	5.611	0.759259	0.745067	0.9506

		B_22_	5	4.073	0.259259	0.2516	0.4066

		N_11_	27	23.131	0.416667	0.925438	<0.001

**R-1994**	48	A_11_	6	5.889	0.680851	0.695264	0.1861

		B_22_	5	4.318	0.276596	0.3578	0.1051

		N_11_	15	15.000	0.30303	0.894173	<0.001

**R-1995**	59	A_11_	6	5.908	0.677966	0.706505	0.4326

		B_22_	3	2.559	0.322034	0.2867	0.0877

		N_11_	23	18.939	0.294118	0.905647	<0.001

**R-1996**	45	A_11_	7	6.912	0.704545	0.731975	0.7935

		B_22_	5	4.463	0.422222	0.3728	0.1599

		N_11_	19	18.591	0.243243	0.912995	<0.001

**R-1997**	48	A_11_	6	5.659	0.770833	0.732017	0.3967

		B_22_	3	3.000	0.4375	0.4070	1.0000

		N_11_	22	20.267	0.333333	0.928283	<0.001

**R-1999**	48	A_11_	6	5.659	0.604167	0.717983	0.3724

		B_22_	6	5.020	0.255319	0.2521	0.3689

		N_11_	20	18.953	0.195122	0.922615	<0.001

^(*) ^H_o _= observed heterozygosity

**^(+) ^**H_e _= expected heterozygosity

**^(x) ^**P_HW_= probability of Hardy-Weinberg equilibrium

Allelic frequencies were remarkably similar between samples; few private alleles, with a very low frequency (average 0.0153), were found. Accordingly, as for mtDNA, the analysis of molecular variance (AMOVA) [23] for loci A_11_, B_22_, and N_11 _was performed both for all samples together and for two separate geographical groups (Table 7). The results indicated that almost all variation (99.74%) was due to within-population differences; subdivision in different populations or different groups was not supported, in agreement with the mtDNA data. Genetic homogeneity detected by the AMOVA analysis was confirmed by pairwise *Fst *analysis (Table 8). In fact, *Fst *values for all pairwise comparisons were negative or close to zero, with associated P-values that were not statistically significant. Similar results were obtained by calculating AMOVA and pairwise Fst without correcting locus N_11 _for null alleles and by excluding locus N_11 _suggesting that the presence of null alleles for locus N_11 _did not affect the analysis of population structure (data not shown). Comparison between the two samples from South Georgia did not show heterogeneity (*Fst *= -0.00120, P = 0.46847), in contrast to the marginal heterogeneity found with mtDNA.

**Table 7:** Analysis of molecular variance (AMOVA) of microsatellite markers.

	Source of Variation	Variance Components	Fixation Index	P value
**One group of 10 populations**	**Among Populations**	0.00294 Va	*Fst*: 0.00299	0.62
	**Within Populations**	0.97999 Vb		

**Two groups** Atlantic Pacific sector (*)	**Among Groups**	-0.00075 Va	*Fct*: -0.00076	0.82
	**Among Populations Within Groups**	0.00330 Vb	*Fsc*: 0.00335	0.53
	**Within Populations**	0.98181 Vc	*Fst*: 0.00259	0.62

AMOVA based on allelic frequencies variation with significance calculated by 10,000 permutations of the original dataset.

^(*) ^Geographical groups: one group with samples from Southern Ocean Atlantic Sector (SG-1994, SG-1997, EI-2007, SS-2007, W-1992, B-1992, P-1997) and the other with samples from the Southern Ocean Pacific Sector (five temporal replicates from Ross Sea. R-1994, R-1995, R-1996, R-1997, R-1999).

**Table 8:** Pairwise *Fst *for microsatellites (below the diagonal) and associated P-values (above the diagonal).

	SG-1994	SG-1997	EI-2007	SS-2007	P-1997	R-1994	R-1995	R-1996	R-1997	R-1999
**SG-1994**	*	0.46847	0.23423	0.61261	0.29730	0.13514	0.30631	0.15315	0.03604	0.98198

**SG-1997**	-0.00120	*	0.64865	0.56757	0.11712	0.34234	0.63063	0.21622	0.29730	0.77477

**EI-2007**	0.00329	-0.00301	*	0.45946	0.13514	0.57658	0.41441	0.62162	0.80180	0.48649

**SS-2007**	-0.00469	-0.00228	-0.00109	*	0.90090	0.50450	0.65766	0.54054	0.17117	0.86486

**P-1997**	0.00145	0.00815	0.00599	-0.00729	*	0.27928	0.30631	0.57658	0.01802	0.51351

**R-1994**	0.00644	0.00199	-0.00333	-0.00170	0.00064	*	0.80180	0.96396	0.20721	0.44144

**R-1995**	0.00185	-0.00325	0.00004	-0.00405	0.00192	-0.00488	*	0.54955	0.09009	0.66667

**R-1996**	0.00774	0.00376	-0.00371	-0.00287	-0.00232	-0.00850	-0.00195	*	0.16216	0.33333

**R-1997**	0.01106	0.00212	-0.00550	0.00444	0.01370	0.00494	0.00890	0.00493	*	0.12613

**R-1999**	-0.00866	-0.00519	-0.00143	-0.00710	-0.00168	0.00007	-0.00328	0.00089	0.00864	*

Pairwise *Fst *based on allelic frequencies variation using locus A_11_, B_22_, and N_11 _with P-values calculated by 10000 permutations of the original dataset; significance threshold α = 0.00111 after multiple test correction.

Estimates of the parameters *θ *and *g *(used to estimate long-term effective population size) are reported in Table 9. These estimates, inferred by a coalescent-based genealogical method [61], were calculated using only locus A_11 _and B_22_, since locus N_11 _showed an allelic pattern that did not follow the stepwise mutation model. Estimates of *θ *among populations ranged from 23.10 for R-1997 (CI = 7.66 - 3,742) to 212.59 for EI-2007 (CI = 34.17 - 10,435). These values were rescaled with the relative mutation rate set to 150; the resulting 95% confidence intervals (CI) were very wide, probably due to the few numbers of loci analyzed. Assuming a mutation rate of μ = 5 × 10^-4^, the long-term *N_e _*value could range from 11,554 (CI = 3,830-1,871,000) to 106,295 (CI = 17,000-5,217,500). Growth rate (g) was also estimated using the same method. The point estimates were positive for all the population samples, ranging from 0.95 for R-1997 to 8.10 for R-1994. The 95% CI excluded zero, except for R-1997 (CI = -0.009 - 7.67) and overlapped between all population samples. Positive values of the g parameter, with CI excluding zero, are an indication that the population has been growing in the past.

**Table 9:** Long-term effective population size and growth based on microsatellite markers.

Population sample	θ	**95% C.I**.	**Ne**^(+)^	**95% C.I**.	g	95% C.I
**SG-1994**	101.31	14.39-5,339	50,655	7,195-2,669,500	7.02	3.00-13.10

**SG-1997**	56.57	13.01-3,049	28,285	6,500-1,524,500	7.66	1.60-11.73

**EI-2007**	212.59	34.17-10,435	106,295	17,000-5,217,500	6.94	2.63-13.69

**SS-2007**	148.12	32.25-9,038	74,060	16,125-4,519,000	6.62	2.36-11.95

**P-1997**	36.38	7.09-428.43	18,190	3,545-214,215	3.20	0.77-10.47

**R-1994**	53.89	13.34-2,981	26,945	6,670-1,490,500	8.10	1.28-13.16

**R-1995**	23.54	7.17-1,792	11,770	3,585-896,000	2.65	0.60-11.34

**R-1996**	78.97	20.02-9,711	39,485	10,000-4,855,500	6.87	1.53-10.73

**R-1997**	23.10	7.66-3,742	11,554	3,830-1,871,000	0.95	-0.009-7.67

**R-1999**	60.58	8.43-2,815	30,290	4,215-1,407,500	6.40	1.46-13.19

Estimates for θ (4N_e_μ, with N_e _= effective population size and μ = nuclear mutation rate per generation) and g (rate of exponential growth) are reported together with their 95% confidence interval (95% C.I.).

^(+) ^N_e _calculated assuming a mutation rate μ = 5 × 10^-4^.

### Forward-time genetic simulations and assessment of statistical power

Since our study was carried out with a limited number (3) of microsatellite markers and a single mtDNA fragment, the statistical power of our dataset was assessed using a simulation approach (Table 10). The power of the 3 microsatellite loci to detect genetic heterogeneity was estimated on the basis of the observed number of loci, observed average allele frequencies, and sample sizes similar to those used in this work. Results showed that the analysed microsatellites reached a power of 0.86 (on a scale of 0-1) to detect an average *Fst *of 0.0045 after 400 generations of pure drift (Table 9). However, for a smaller extent of drift, or assuming one to five migrants per generation (values that define a "strict" or "relaxed" genetic threshold for isolated populations according to Waples and Gaggiotti [68]), the percentage of rejections of the null hypothesis of genetic homogeneity dropped considerably (Table 9). This holds true also for values of *Fst *close to those observed in our empirical microsatellite dataset (i.e., 0.00299, Table 7). When considering simulations based on a mitochondrial dataset, positive values of *Φst *are expected (on average) in all scenarios, which is in sharp contrast to the experimental results obtained from the analysis of ND1 sequences (*Φst *= -0.0023; Table 3). Simulations obtained from a combined microsatellite + mtDNA dataset (possible in virtual simuPOP by jointly modelling haploid sequences and diploid microsatellite genotypes) clearly showed that a statistical power higher than 80% to detect genetic heterogeneity with at least one marker was achieved after 300 generations of divergence under all scenarios. These results suggest that our markers can provide an accurate picture of the large scale pattern of genetic population structure of *E. superba*, unless the differentiation is more recent than 300 generations.

**Table 10:** Power to detect differentiation using datasets simulated with simuPOP ver 1.0.0

Nm	Gen	μsat mean Fst	μsat power	mtDNA mean Fst	mtDNA power	Combined power
0	100	0.00123	0.22	0.00378	0.18	0.36
0	200	0.00271	0.34	0.00916	0.55	0.64
0	300	0.00272	0.62	0.01691	0.88	0.96
0	400	0.00445	0.86	0.01988	0.94	1.00

1	100	0.00131	0.18	0.00731	0.22	0.38
1	200	0.00222	0.44	0.00843	0.44	0.76
1	300	0.00291	0.68	0.01466	0.7	0.92
1	400	0.00383	0.84	0.01483	0.82	0.98

5	100	0.00115	0.22	0.00532	0.22	0.36
5	200	0.00147	0.28	0.00868	0.35	0.50
5	300	0.00211	0.36	0.01252	0.66	0.80
5	400	0.00255	0.52	0.01372	0.76	0.90

Ten populations of individuals with 3 diploid loci and one sequence haplotype, initialized with the empirical microsatellites and mitochondrial dataset, were simulated and allowed to diverge with a predefined number of migrants (Nm) for a fixed number of generations (Gen) before data collection and analysis with AMOVA. For each set of parameters one hundred replicates were performed. Reported are the mean Fst recorded and the percentage of dataset providing significant differences across replicates for microsatellites (μsat mean Fst and μsat power) and mitochondrial DNA (mtDNA mean Fst and mtDNA power). In addition the percentage of cases in which at least one marker detected significant differences is reported in the last column.

## Discussion

The present study is aimed at investigating the levels of genetic variation and historical demography of *E. superba *using both mitochondrial DNA and microsatellite markers for an unprecedentedly comprehensive sampling of populations over a circum-Antarctic range.

Analyses of species with putatively weak population genetic structure such as *E. superba *should be ideally conducted by means of hyper-variable markers such as microsatellites, suitable to detect subtle genetic differences between populations. This approach proved problematic in krill, due to low efficiency of the markers' isolation protocol and redundant genomic sequence portions. Considerable effort was made to isolate nuclear DNA microsatellite markers specific to *E. superba *using the FIASCO protocol [41]. However, this resulted in an extremely limited number of loci containing microsatellites. In addition, a large portion of the isolated loci were characterized by complex structure that made them unsuitable for population genetics studies [32], suggesting that Antarctic krill may have some peculiarity in genomic organization and/or composition. Given these difficulties, we used a specific ESTs dataset for *E. superba *[40] to identify EST-linked microsatellites. This approach yielded a limited number of microsatellites suitable for population genetic analysis. Again, many loci showed multiple alleles per individual, incompatible with simple Mendelian inheritance. Sequences were characterised by long stretches of Thymine (T) and Adenine (A), in keeping with old reports of strong A/T bias in base composition in krill genome [31].

Despite these limitations, the few microsatellites used in this work provide important support and new perspectives that complement the results obtained from mitochondrial sequences. In fact, the forward time simulation approach, which represents to our knowledge the first attempt to jointly model nuclear and mitochondrial markers in a population genetic context for a non-model organism, shows that the present dataset is endowed with considerable power to detect genetic heterogeneity, because of very large sample size and despite the low number of loci considered. We can, therefore, confidently accept the results from the mtDNA and nuclear empirical dataset, which indicate small, not significant, genetic differences among samples and pinpoint panmixia of Antarctic krill populations on a large geographic scale, but leave open the possibility for local genetic patchiness. Neutrality test associated with analysis of mismatch distribution carried out for the ND1 mitochondrial gene revealed strong signals of past population expansion. This was confirmed by positive values of the parameter, *g*, a measure of population growth obtained from microsatellite markers with a pattern that further supports a recent origin of the population expansion.

### Lack of genetic heterogeneity on the circum-Antarctic scale

Comparison of mitochondrial ND1 sequences among 12 populations from the Atlantic and Pacific sectors of the Southern Ocean showed a lack of genetic structuring, as suggested by negative or not significant values of *Fst*. Similarly, analysis of molecular variance based on 3 microsatellite loci did not reveal significant genetic differentiation among populations. These results are indicative of high levels of gene flow over a large geographic range, preventing the formation of genetically isolated gene pools. Although the absence of statistically significant differences cannot be equated with proof against genetic structure, the statistical power of our dataset, estimated by the forward simulation approach, suggests that the level of genetic divergence must be exceedingly small to have remained undetected (Table 10). This result is somewhat counterintuitive, considering both the small number of microsatellites and the very short ND1 sequence used; it is due to the large sampling used in this study and to the very high number of haplotypes found at the ND1 locus. In particular, 29% (46 of 154) of the ND1 sites were variable - very close to the 33% of third positions available in the dataset. This can result in a potential saturation problem that is expected to increase the chances of obtaining haplotypes identical-in-state but not by descent, thus blurring any eventual phylogeographic pattern. However, the very high number of haplotypes results in a high chance to detect differences in haplotypic frequencies, as evidenced by our simulation approach (Table 10), thus partially compensating the problem.

We can speculate about the causes of such genetic homogeneity and several reasons can be invoked to explain our finding. The circum-Antarctic current system may play an active role in mixing krill populations, thus favouring the maintenance of a single randomly-mating unit. In fact, the entire life history of *E. superba *is influenced by the Southern Ocean current systems: females are thought to perform a spawning migration in deep water, eggs are laid in the eastward-flowing ACC current, embryos perform a "developmental ascent" migrating upward, and juveniles spend the winter under the ice that is drifted by the currents [[69,70], but see also [71]]. The lack of large scale genetic structuring might then simply reflect the tight link between krill and circum-Antarctic marine currents.

### Genetic patchiness at the local scale?

A previous study by Zane et al. [36] found significant mtDNA genetic heterogeneity among samples collected from the Weddell Sea and near South Georgia; the authors suggested that the Weddell-Scotia Confluence and the Weddell gyre could act as oceanographic barriers, leading to genetic differentiation between populations in the two regions. In the present paper, we increased the number of analysed samples from 249 specimens in four population samples [36] to 642 specimens in 12 population samples (including temporal replicates). The results obtained by this expanded dataset confirmed the weak differentiation between Weddell Sea and South Georgia 1994 (*Φst *= 0.02130, P = 0.033), samples shared with the Zane et al. [36] dataset, but significance was not achieved after correction for multiple tests due to the larger number of samples considered. In addition, a similar value of *Φst *was also observed between the two replicated samples collected during 1994 and 1997 from South Georgia (*Φst *= 0.02438, P = 0.029), ruling out the possibility that the former result can be explained by the presence of stable geographically isolated populations.

The biological meaning of these subtle differences is uncertain, because the differences reported here are very small and not significant after multiple test correction, and thus could simply represent false positives. On the other hand, they can reflect biological or physical processes at other spatial and/or temporal scales, making the correction procedure inappropriate. In fact, inter-annual variation of krill biomass due to variable recruitment rates is well known in the South Georgia region [72] and, based on oceanographic modelling, it has been suggested that recruitment in this area relies on reproduction in the Antarctic Peninsula [73]. Recent observations indicate the presence of multiple spawning and larval development sites in the West Antarctic Peninsula [71,74] and several factors can affect the successful transport of krill to the S. Georgia area, including the location and timing of spawning and the annual variation of the position of the Southern Antarctic Circumpolar Current Front (SACCF), potentially leading to random variation in the genetic composition of local recruits [75]. A recent independent study based on Cytochrome *b *SNPs (Single Nucleotide Polymorphisms) analysis of different life stages collected in the Western Antarctic Peninsula [37] provides strong support for this hypothesis. In fact, while no overall differences were found between 2001 and 2002 collection years, significant differences occurred among samples from the same area collected in the same year, and differentiation was particularly evident among samples when furcilia larval stages were analysed separately [37]. These results indicate a stage-specific patchiness that might result from the advection and persistence of cohorts of larvae from diverse spawning sites or from discrete spawning events, if they involve a limited number of breeders. In fact, fine-scale genetic patchiness can result from temporal variation of numbers and genotypes of recruits delivered locally [75] and it is commonly believed to arise from instantaneous drift effects [76] for which the genetic pool of recruits can change from time to time due to random effects. Following Hedgecock's [77] hypothesis of sweepstakes reproductive success, temporal variance in allelic frequencies may result from stochasticity in reproductive activity and oceanographic conditions conducive to fertilization, larval development, retention and recruitment. Under this hypothesis, many individuals fail to contribute to recruitment and this variance may result in changes in allelic frequencies when differences in allelic composition among spawning groups are present. Hedgecock [77] attributed the variation in reproductive success of adults to spatio-temporal variation in oceanographic conditions that occur within and among seasons. Considering that genetic patchiness does not require stable genetic differentiation of populations and unpatterned variation has indeed been found in many noticeable cases in species with very weak differentiation on the large scale [i.e. [78-81]], it is possible that this phenomenon may occur in Antarctic krill, as suggested by Batta-Lona et al. [37]. This finding is not in conflict with the lack of differentiation at large geographic scales found in our study. From this point of view, weak but recurrent temporal or small-scale differences observed for krill [33-35] might result from oscillations associated with recruitment processes. Multi-year investigations that incorporate environmental data and focus on krill populations from areas with high variation in recruitment, such as S. Georgia, are therefore needed before dismissing small-scale heterogeneity as "ecologically uninformative" [82].

### Strong and recent population expansion in krill

Analysis of the mtDNA ND1 fragment for 642 samples confirmed its high sequence polymorphism, with 124 haplotypes observed and an average value of π = 0.013. This variability is particularly high considering the length of the fragment (154 bp) and comparing the variability of a 155 bp fragment of the same gene in *M. norvegica *(35 haplotypes over 982 individuals, π = 0.005) [83]. Jarman et al. [39] found 82 haplotypes among 232 individuals for a mtCOI gene segment of 665 bp in *E. crystallorophias*. High polymorphism of the ND1 mitochondrial gene portion for *E. superba *could be ascribed to the departure from the mutation-drift equilibrium, resulting in the retention of rare haplotypes. In fact, haplotype diversity (h = 0.85) indicates that about 85% of the haplotypes occur only once in the entire dataset. The hypothesis of departure from mutation-drift equilibrium is suggested also by the haplotype network reconstruction, which reveals a star-like tree topology. Rare haplotypes are derived from frequent ones by few substitutions and the tree topology is thus compressed near the root [54]. High numbers of rare haplotypes - leading to the departure from mutation-drift equilibrium - is a clear indication of past population expansion. This hypothesis is also supported by the concordant results of the neutrality tests that allow rejection of the mutation-drift equilibrium hypothesis with high confidence. An alternative explanation that selection acts on mtDNA sequences and causes departure from equilibrium seems unlikely, considering that only 3 substitutions are non-synonymous over 59 substitutions recorded. Variation of population size better explains the non-equilibrium condition of *E. superba*, as suggested by the analysis of mismatch distribution. The distribution of pairwise differences between haplotypes was a better fit to the theoretical distribution curve for a population that experienced a demographic expansion, peaking approximately at τ = 2.50. This value is higher than that obtained by Zane et al. [36] based on fewer samples (τ = 1.66).

If accurate estimation of the substitution rate *u *was possible, the peak of the curve (τ) could be related to the time at which the population expansion started. Unfortunately, no calibration of rates is possible for pelagic organisms lacking fossil records (such as euphausiids) and the use of previously-calculated "universal" rates [36] is not straightforward, due to rate variation among lineages (see e.g., [84]). In addition, it has been recently shown that rates calibrated in a phylogenetic context should be applied with caution to intraspecific studies, because this could result in over-estimation of divergence times and expansion peaks, since many of the polymorphisms seen at the population level are lost over longer evolutionary time frames [85]. As a matter of fact, importing the crustacean phylogenetic universal rates as in [36] to the present dataset would result in a time of start for krill expansion ranging between 0.1 million to more than 1 million years (taking into account the confidence interval of τ). This time window is concordant with recent results based on a Bayesian skyride approach, using a different marker and a slightly different universal rate [86], which showed that the most recent doubling in abundance occurred within the last 100 000 years [38]. Given the possible overestimation due to time dependency of these values, which could be as high as 10-20 fold older than the correct ones [85], it is conservative to consider them as maximum ages for expansion, bearing in mind that they could be close to the true value in the case of little or no time dependency of the molecular clock calibrations [87,88].

A specific estimation of divergence rate for euphausiids could be the only way to address the problem of incorrect calculation of population expansion time and to allow useful inference about the effect of paleoclimate on krill population dynamics. This aspect is of particular interest when considering that the Antarctic region has experienced important climatic changes in the last 740 000 years, including temperature fluctuations ranging between 8 to 10°C, with a cyclic pattern of about 100 000 years [89]. These fluctuations promoted migrations of the marine fronts northward and southward, re-shaping the boundaries of the Southern Ocean and probably affecting population dynamics of Antarctic pelagic species such as Antarctic krill. At present, based on the mtDNA marker, we can only point out that, given the possible overestimation in divergence times, our value of τ could in principle be very recent and potentially generated by expansion during the last glacial maximum, when Antarctic planktonic ecosystems migrated equator-ward [90], in contrast to the picture reported before for krill [36] and other Antarctic marine species [91,92].

Microsatellite analyses confirmed that Southern Ocean krill experienced a strong population expansion in the past. Growth rate estimates were positive for all population samples and the associated confidence interval was significantly higher than zero in nine of ten cases. Non significant differences between growth rates were observed between samples (Table 10), which could reflect distinct trajectories of growth linked, for instance, to possible undetected genetic heterogeneity at the local scale. However, considering that the growth rates reported, estimated by the Bayesian approach implemented in LAMARC, refer to overall growth during the coalescent time in which polymorphisms accumulated in the populations, it is more reasonable to assume that such insignificant differences are simply linked to sampling errors. Thus, the approach used here is unable to appropriately detect subtle differences in growth or, since it is based on a simple exponential model, frequent population changes.

On the other hand, LAMARC allows calculation of theta (*θ) *and estimation of the effective population size using the exponential decay equation *θ_t_=θ_now _e^-gt ^*with time toward the past expressed in mutational units [61]. Conservatively using the highest estimated value of theta (212.59) and the smallest rate of growth (0.95; see Table 9), krill populations would have dropped 90% in the past 5,000 generations and have collapsed to as few as 10 effective individuals in less than 20,000 generations. Thus, if the values estimated by LAMARC are realistic and assuming a 2-3 year generation span [36,93], exponential growth (or decline backward in time) may have occurred no longer than 60 000 to 40 000 years ago. Using average values for *θ *and g (Table 10), krill population expansion may be as recent as a few thousands years.

## Conclusions

The results reported in this work provide strong evidence for lack of genetic heterogeneity of Antarctic krill at the large geographic scale and unequivocal support for past population growth. The lack of genetic structuring might reflect the tight link between krill and circum-Antarctic Ocean currents, whereas small-scale spatial and temporal differentiation might be due to local conditions. Apparently, no geographical barriers limit the gene flow among different areas. Since gene flow in marine species is largely due to dispersal capabilities of larval phases, it is expected that species with high dispersal potential, such as *E. superba*, may exhibit low levels of population differentiation [94], whereas species with limited dispersal and/or subject to geographical barrier may show a significant degree of genetic structuring [95]. In the case of Antarctic krill, both mitochondrial and nuclear markers point toward an overall panmictic condition, consistent with the hypothesis that differentiation processes in Antarctic species is largely influenced by dispersal potential. The signal of past population growth suggests the differential impact of glacial cycles on pelagic Antarctic species, for which glaciations possibly boosted population expansions due to the increase of available habitat, in contrast to benthic sedentary species, for which they are believed to have promoted differentiation due to local extinction and post-glacial re-colonization.

From a management perspective, it is important to recognise that lack of genetic differentiation does not imply that krill populations should be managed as a single stock, since the amount of migration needed to maintain populations genetically homogeneous is far smaller than the amount required to make them demographically undistinguishable. Using the threshold defined by Waples and Gaggiotti [68] to define populations based on an ecological paradigm, up to 10% of individuals can be exchanged each generation among populations that maintain their demographic independence, resulting in a maximum number of about 1,000-10,000 effective migrants (Table 9). This level of exchange cannot be excluded with our approach (Table 10) and would require many more markers and sampling to directly estimate demographic connectivity between locations [96,97].

Our study shows that microsatellite identification by data mining of EST libraries is a powerful approach to avoid failures associated with conventional enrichment techniques in this species. However, considering our low success rate (2 loci without null alleles from 68 candidates identified from 1,170 ESTs obtained with Sanger sequencing, which in our opinion confirm some peculiarity of the krill genome), the isolation of a suite of hypervariable markers for this species will require a concerted and comprehensive sequencing effort using high throughput technologies.

## Authors' contributions

EB carried out the molecular genetic analyses. EB and LZ analyzed the data. MM performed forward-time simulations. TP conceived the study and coordinated the research. EB, LZ and TP wrote the paper. AB participated in the design of the study, discussed results and contributed to drafting the manuscript. All authors read and approved the final manuscript.

## Supplementary Material

Additional file 1 **Table S1**. Distribution of ND1 haplotypes in the populations. Click here for file

Additional file 2 **Table S2**. Characteristics of the 12 microsatellite loci identified from EST sequencing [40]. Click here for file
