# Supplementary material for: Gone with the currents: lack of genetic differentiation at the circum-continental scale in the Antarctic krill Euphausia superba
Source: BMC Genet. 2011 Apr 12;12:32. doi: 10.1186/1471-2156-12-32 (PMC3095564; doi:10.1186/1471-2156-12-32)
Supplement: Additional file 2 — Table S2. Characteristics of the 12 microsatellite loci identified from EST sequencing [40]. [file 1471-2156-12-32-S2.DOC]

**Table S2 - Characteristics of the 12 microsatellite loci identified from the cDNA library.**

| EST sequence | Primer name | Primer sequence (5′→3′) | Successful primer pair | Anneal. Temp. | Dye | Polymorphic Loci | Amplif. Pattern | Loci Used |
| --- | --- | --- | --- | --- | --- | --- | --- | --- |
| Kr 06_02C03 | A_1_ for | TTCCCCTGTGGAAGAAGATG | A_11_ | 55°C  35 cycles | TAMRA | √ | diploid | √ |
|  | A_2_ for | TGGAGAGCTGTGTGGAAGAG |
|  | A_1_ rev | GCCTGTGCTTTCTTCTCTGG |
| Kr 05_03E12 | B_1_ for | AACTATGGAGGGGGTGGGCAC | B_22_ | 55°C  35 cycles | HEX | √ | diploid | √ |
|  | B_2_ for | TCTAAGCCTGTTCTTGGGTCA |
|  | B_1_ rev | TGGTTTCTCTATAACTGGTGCC |
|  | B_2_ rev | GTTGTCCTTGCTTTTCTTCCAG |
| Kr 06_02F10 | C_1_ for | ATCTCATGCCACCACCACCA | - | - | - | - | - | - |
|  | C_1_ rev | GCGGGGCATATAAAGAAGATG |
|  | C_2_ rev | TAATATGACAACAGCGGGGCA |
| Kr 06_01H09 | D_1_ for | TGAAATTAAGTGATACCACAAC | D_11_ | 55°C  30 cycles | HEX | - | - | - |
|  | D_2_ for | CCCTCTATGACATGATTGGTC |
|  | D_1_ rev | GAAGTTTGAAGGCTTACTGGC |
|  | D_2_ rev | CGTAACCTCACTCGTTCACTG |
| Kr 09_02K02 | E_1_ for | GACTGGTCTGCTGATGGTCA | - | - | - | - | - | - |
|  | E_2_ for | CCGGATTCCAGGTTACTGAA |
|  | E_1_ rev | TGTCACTACCACTGGGTGGA |
|  | E_2_ rev | CTGTCTGGCCACATCAACAG |
| Kr 09_02G08 | F_1_ for | TTGCCACAGCTTGTTTTGCC | F_12_ | 55°C  30 cycles | FAM | - | - | - |
|  | F_2_ for | TGTGGTAATGCTTATCATTGG |
|  | F_1_ rev | TATTCAAATAACACCCACGGTT |
|  | F_2_ rev | CACATTTTATTCAAATAACACCC |
| Kr 05_01B10 | G_1_ for | TTCCATCTTCACACACATTGC | - | - | - | - | - | - |
|  | G_3_ for | ATGACGAGGGCAGAATATGC |
|  | G_1_ rev | TGCGGCTGCTTTGACTGACA |
|  | G_3_ rev | ATACAAAAGAATATGGGCGAC |
| Kr 01_01E01 | H_1_ for | GTTGGAAAGTGATAAGTGTT | H_12_ | 52°C  35 cycles | FAM | √ | non-diploid | - |
|  | H_2_ for | CAGCATCATACCTGCAAGC |
|  | H_1_ rev | TATAAACATTTCAAAGTGAATTA |
|  | H_2_ rev | TCTACTTTAATATATATGTTCCA |
| Kr 07_03L20 | I_1_ for | ACAGATGTCAGGGAGAAGGTTG | - | - | - | - | - | - |
|  | I_2_ for | CAGGGAGAAGGTTGACAGCGAT |
|  | I_1_ rev | CTCTGTTTCCCATCTCGGTTTC |
|  | I_2_ rev | GTACCTTCTTCATTGACACTGT |
| Kr 06_02O11 | L_1_ for | CCTTTCTACCTTGGTGAGGT | - | - | - | - | - | - |
|  | L_2_ for | GTCCAACCAATCCAACGGCA |
|  | L_1_ rev | TCCACTAGCGTCATCACCAC |
|  | L_2_ rev | ATTTTCTTCTTCCACTAGCGTC |
| Kr 06_02M15 | M_1_ for | GGCGTTGGCTCAGATGTCG | M_11_ | 55°C  35 cycles | TAMRA | √ | non-diploid | - |
|  | M_2_ for | GTCACCGACACCACCGTG |
|  | M_1_ rev | ACCTCTCTAGTCTTGGATCAG |
|  | M_2_ rev | TATCAATTCCAATCGCTCTGC |
| Kr 06_02E21 | N_1_ for | ATAAATCGCCCGTTTGACGA | N_11_ | 52°C  35 cycles | HEX | √ | diploid | √ |
|  | N_3_ for | AATCGGACCATAAATGTGACCT |
|  | N_1_ rev | ATTGAGGGGATATTTGGTAGGA |
|  | N_2_ rev | TTGATACATATATTGAGGGGAT |
